# Predictive value of a diagnostic block in focal nerve injury with neuropathic pain when surgery is considered

**DOI:** 10.1371/journal.pone.0203345

**Published:** 2018-09-12

**Authors:** Martijn J. A. Malessy, Ralph de Boer, Ildefonso Muñoz Romero, Job L. A. Eekhof, Erik. W. van Zwet, Michel Kliot, Albert Dahan, Willem Pondaag

**Affiliations:** 1 Department of Neurosurgery, Leiden University Medical Center, Leiden, The Netherlands; 2 Neurological Center at the American British Cowdray Medical Center, Mexico City, Mexico; 3 Department of Neurology, Alrijne Hospital, Leiden, The Netherlands; 4 Department of Statistics, Leiden University Medical Center, Leiden, The Netherlands; 5 Department of Neurosurgery, Stanford University School of Medicine, Stanford, California, United States of America; 6 Department of Anaesthesiology, Leiden University Medical Center, Leiden, The Netherlands; University of Würzburg, GERMANY

## Abstract

**Object:**

In patients with focal nerve injury and neuropathic pain cutting the nerve to obtain permanent pain reduction can be considered. Surgery is indicated only if a diagnostic nerve block provides temporary pain relief. We evaluated the predictive value of a block on the outcome of surgery.

**Methods:**

In total, three blocks were performed at two week intervals. Patients were blinded to injections containing lidocaine 1% and a placebo was included. Surgery was offered regardless of the effect of the blocks. Twenty-four patients received 72 blocks. Sixteen patients opted for surgery, 5 patients refrained from surgery, and in 3 the blocks provided permanent pain relief. The predictive ability of the block on the outcome of surgery was assessed by calculating the area under a Receiver Operating Characteristic curve (AUC).

**Results:**

The AUC of the first lidocaine block was 0.35 with a 95% confidence interval from 0.077 to 0.62. At 95% confidence (two-sided), the AUC is less than 0.62, and hence the predictive ability of the block was poor. The outcome of the second lidocaine block and saline block did not change the conclusion of the first block.

**Conclusions:**

We conclude that the use of blocks to select patients for surgery should be critically appraised.

**Perspective:**

A pain relieving response to one open block is currently considered mandatory before patients with focal nerve injury and neuropathic pain are offered surgery. Blinded blocks including a placebo show that responses for selection should be carefully interpreted because they may not be as predictive as generally presumed.

## Introduction

Chronic pain of moderate to severe intensity occurs in 19% of adult Europeans, 4% of which is caused by nerve damage.[1,2] The neuropathic pain that arises from nerve damage is associated with abnormal sensation or hypersensitivity in the area of the affected nerve, which can be adjacent to or combined with areas with reduced sensation. Patients experience paraesthesia (*i*.*e*., skin crawling sensation or tingling), spontaneous (not stimulus-induced) ongoing pain, and shooting, electric shock-like sensations.[3] Neuropathic pain due to a nerve lesion is difficult to treat. Only a minority of patients have an adequate response to pharmacotherapy.[3,4] Alternatives such as neurostimulation therapy are increasingly applied, but their role needs yet to be defined. So far, most trials on neurostimulation for pain relief did not comply with the requirements of evidence-based medicine.[5]

**Fig 1:**
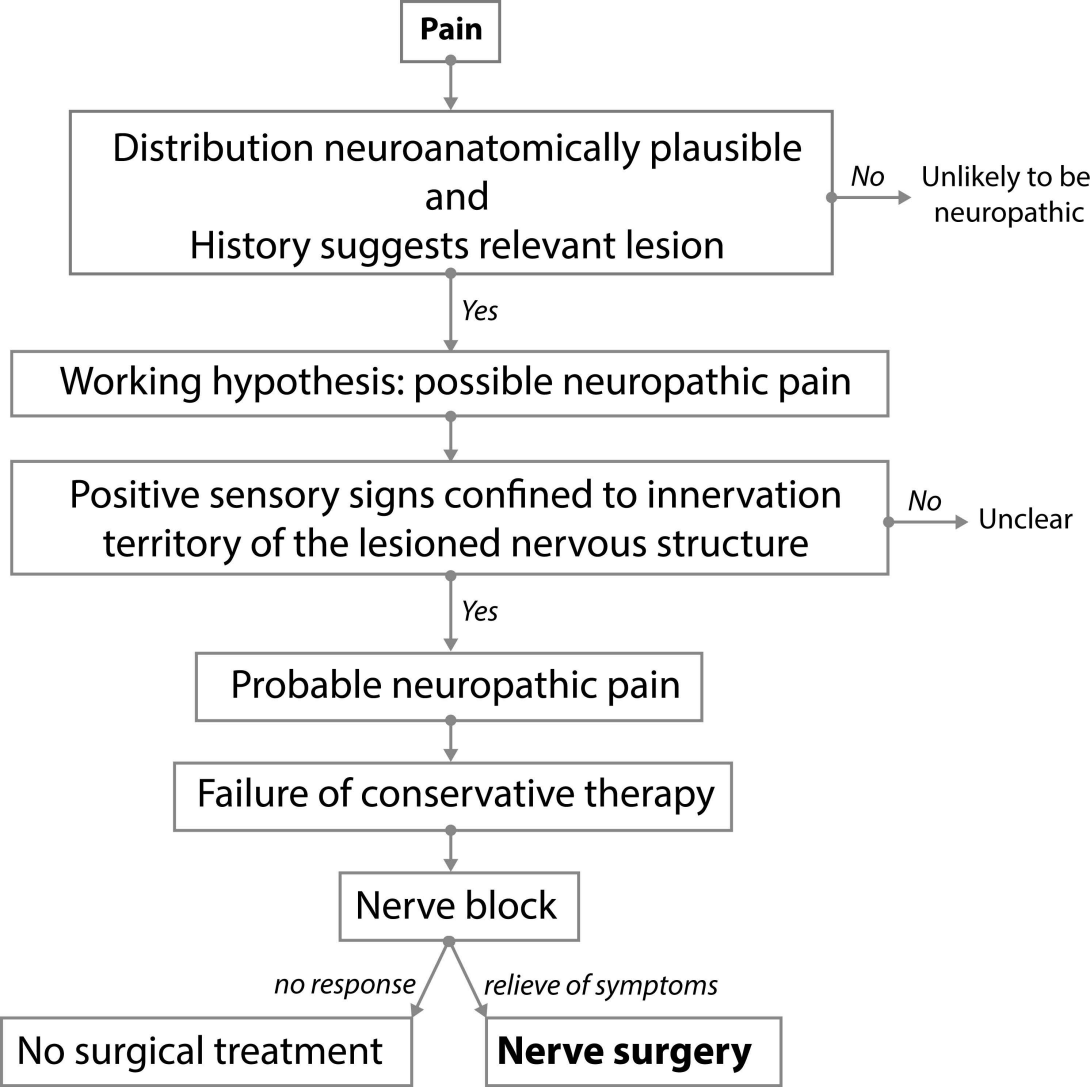
Current selection algorithm for nerve surgical treatment of patients with neuropathic pain due to a focal trauma to a nerve. The decision whether to operate or not is based on one “open” nerve block. Adequate temporary pain relief following the block is considered mandatory before surgery is undertaken. The nerve is then cut proximal to the painful area to obtain permanent pain reduction.

Surgical treatment of the damaged nerve is considered as an option when all other treatments have failed. The surgical technique consists of either neurolysis and releasing the nerve from the scarred area or neuroma resection with relocation and burying of the proximal stump.[6] The nerve innervating the area of pain can be identified by assessing the area of sensory abnormality, tapping over the course of the nerve and by blocks with local anaesthetics.[7] Nerve blocks are also used in the decision-making process to determine whether to perform a surgical intervention. Adequate temporary pain relief following the block is considered mandatory before surgery is undertaken.[7–55] [56–62] (Fig 1) There is no objective evidence base for blocking a nerve before nerve surgery is undertaken. Only one study assessed the predictive value of a diagnostic nerve block for the pain relieving effect of surgery. However, patients who did not respond to the block were not operated thereby introducing a selection bias.[52] The weakness of the currently used algorithm appears that the decision whether to operate or not is based on just one “open” block that might be effective due to an inherent effect on the damaged nerve or that may be contaminated by a placebo effect.

**Fig 2:**
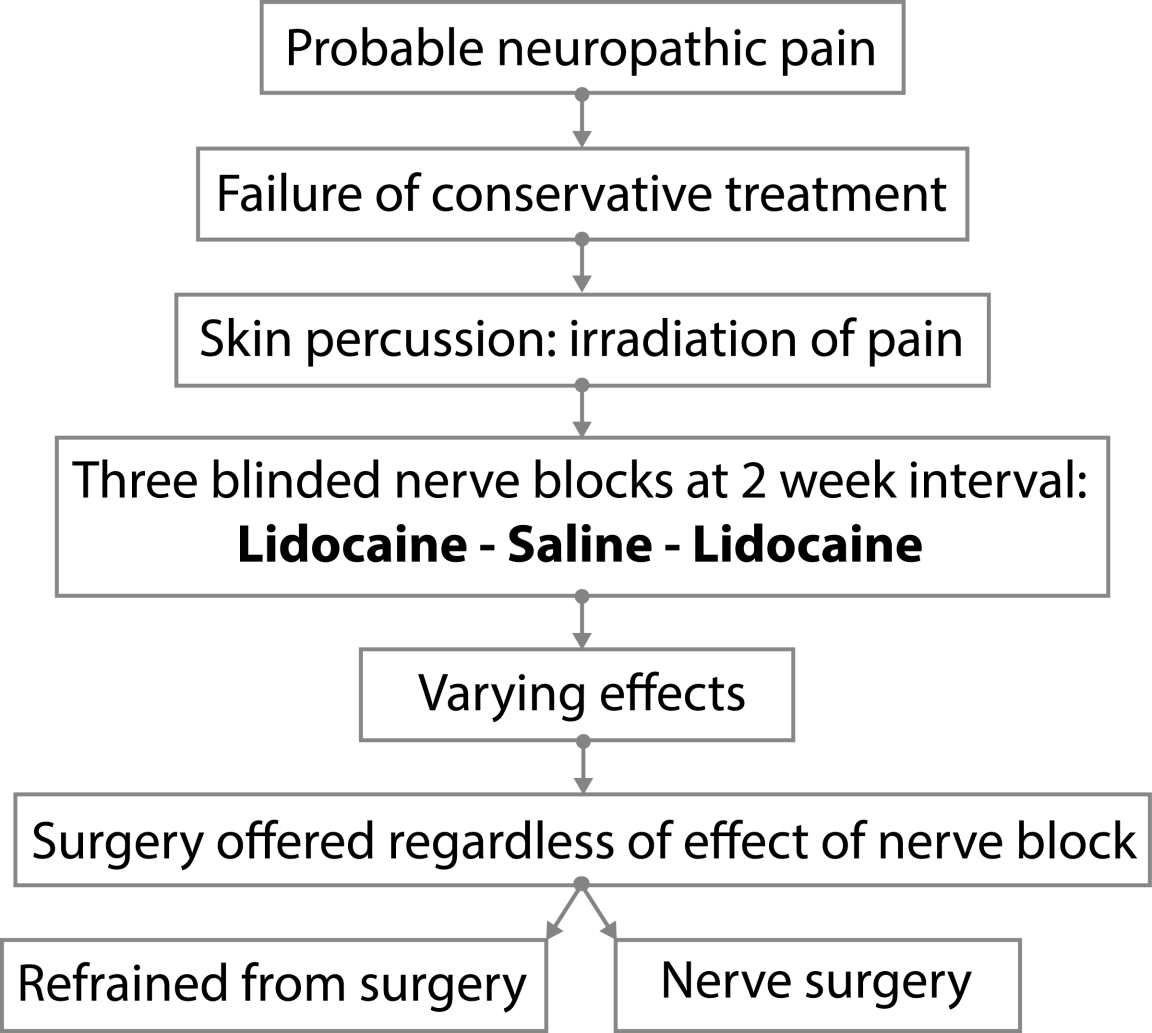
The LUMC protocol applied exclusively to patients with persistent postsurgical or nerve injury–induced probable neuropathic pain related to sensory nerves. All patients had positive sensory signs confined to the innervation territory of the damaged nervous structure and had insufficient pain reduction or severe side effects from pharmacotherapy. The involved nerve is blocked three times in 4 weeks (with a 2-week interval between injections) and consists of two single-blind injections with lidocaine and one with placebo. All patients are given the opportunity of surgery irrespective of the outcome of the block injections.

We used a different protocol in our patients. The protocol is applied exclusively to patients with probable neuropathic pain due to nerve injury[2] and who had insufficient pain reduction or severe side effects from pharmacotherapy. The involved nerve is blocked three times in 4 weeks (with a 2-week interval between injections) and consists of two single-blind injections with lidocaine and one with placebo.(Fig 2) All patients are given the opportunity of surgery irrespective of the outcome of the block injections.

We looked for the level of evidence for the use of a block for selection (Fig 1) by assessing the predictive value of a block on the outcome of surgery.

## Methods

### Leiden nerve block protocol

A standardized diagnostic nerve block protocol was applied in the work-up of patients in whom nerve surgery to treat neuropathic pain was considered.(Fig 2) All of these patients had had extensive conservative pain treatment prior to referral, which was insufficient or induced significant side effects. For this study, only patients were included who had persistent postsurgical or nerve injury–induced neuropathic pain related to sensory nerves and who had positive sensory signs confined to the innervation territory of the damaged nervous structure. Prior to the block, the area with aberrant skin sensation (allodynia, hypaesthesia, dysaesthesia) was assessed. The involved nerve and site of maximum pain provocation was anatomically localised by percussion from distal to proximal over the course of the suspected nerve and scar. Neuroma provocation was considered present when tapping increased the pain and provoked tingling in the area with aberrant skin sensation. In total, three injections were given at two-week intervals. Patients remained blinded for the nature of the injections (active or placebo). No information was provided about the potential effects or duration of effects. The first and third injections were performed with lidocaine 1%; the second injection with placebo (water Sodium Chloride 0.9% solution, saline); each injection had a volume of 4 cc. All injections were performed by the same surgeon with a vast experience in nerve surgery. The site of injection was assessed by tapping from distal, starting in the area with the sensory deficit, to proximal, over the anatomical trajectory of the damaged nerve. The injection was positioned just proximal to the area of maximal pain provocation upon tapping. The direction of the needle and depth of the tip was repositioned several times in order to circumvent the nerve and provide an adequate infiltration as regarding to depth and size of the area of the involved nerve. After removing the needle, pressure was applied to improve the spread of the fluid. All three injections were performed at the same location. Two weeks after the injection the response to the injection was documented and arranged in four groups: 0 = no effect of the injection; 1 = pain relief for several hours; 2 = pain relief for days or permanent pain relief; 3 = increase of pain for hours or days. Two weeks after the third block, the content of all three provided injections was revealed to the patient and the effect of the injections was subsequently discussed. The patients were informed about the current treatment paradigm (Fig 1) implying that surgery is offered when the response to lidocaine was positive. Regardless of the outcome of the three blocks, all patients were offered nerve surgery. All patients provided verbal informed consent. The Leiden University Medical Center Institutional Review Board declared that ethical approval was not required for this study.

### Nerve surgery

Patients were operated under general anaesthesia. The nerve trunk proximal to the area of maximal pain was dissected free and dissection continued distally into the zone of tissue and nerve damage. The responsible nerve branch ending was identified and cut just proximally to the neuroma into healthy looking nerve. Skin branches leaving the nerve trunk proximal to the neuroma and which were not involved in the lesion were left intact. The neuroma of the damaged nerve was resected. The proximal nerve stump was loosely buried as deep as possible away from potential sites of mechanical pressure and joints. The stump was enveloped by fat tissue and secured with a 5.0 vicryl suture. Finally, wound closure was performed according to standard procedures. The effect of surgery on pain was documented using five point scale: 0 = no effect, 1 = partial pain reduction, 2 = (as good as) pain free, 3 = temporary pain reduction, 4 = increase of pain as compared to the pre-operative level.

### Data analysis

To assess whether the response to the nerve block is predictive of the success of the surgery, we proceeded as follows. The result of the block was ordered according to a (presumed) increasing likelihood of a successful surgery: a) no effect; b) increase of pain for hours or days; c) pain relief for days; d) pain relief for several hours. The outcome of surgery was dichotomized as unsuccessful (those surgeries with no effect, or temporary pain reduction, or an increase of pain as compared to the pre-operative level) or as successful (partial pain reduction or (as good as) pain free following surgery). A decision rule for recommending surgery would involve a cut-point for the result of the block. For instance, one might recommend surgery to patients who score better than b) on the block. Such a decision rule will result in two types of errors: recommending surgery to some patients who do not benefit, and not recommending surgery to patient who do benefit. Changing the cut-point will impact the probabilities of both types of errors. Plotting the sensitivity versus 1-specificity of the decision rule for varying cut-points yields a so-called Receiver Operating Curve (ROC). The area under this ROC cure (AUC) is a commonly used measure of predictive ability. The AUC is expressed between 0 and 1. The predictive ability and accuracy of a diagnostic block is excellent if the AUC is between 0.90–1.0. The test is good with an AUC between 0.80–0.90, fair between 0.70–0.80, poor between 0.60–0.70, and failed if the AUC is between 0.50–0.60. An AUC less than 0.5 is worse than random guessing.[63;64] We used a Bootstrap test with 10000 replications as implemented in the R package pROC[65] for correlated ROC curves to assess whether the first lidocaine block differs from the second lidocaine block.

## Results

**Fig 3:**
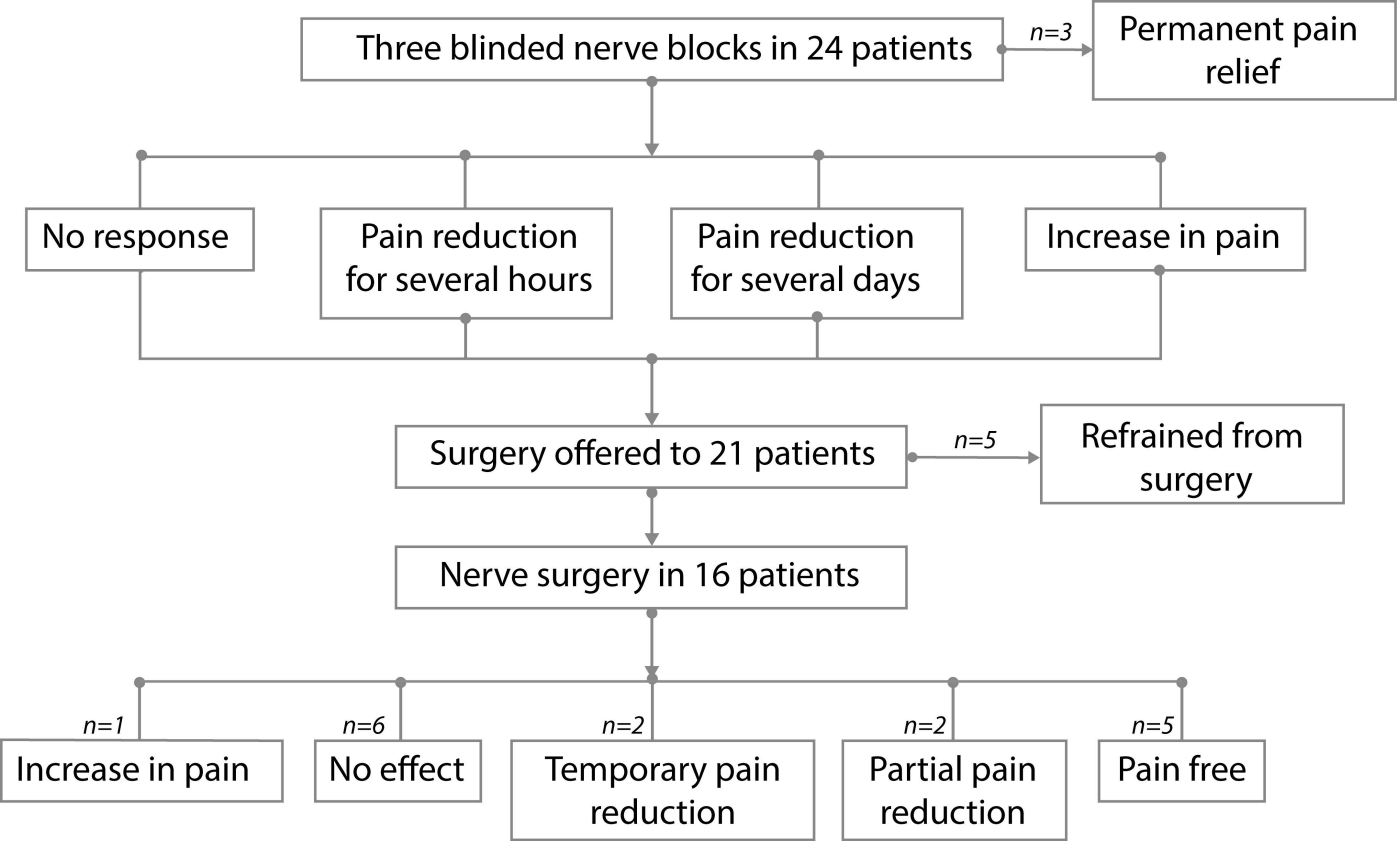
The effect on pain of three blinded nerve blocks in 24 patients with persistent postsurgical or nerve injury–induced probable neuropathic pain related to sensory nerves and results of nerve surgery. Two weeks after each injection the response to the injection was documented. Two weeks after the third block, the content of all three provided injections was revealed to the patient and the effect of the injections was subsequently discussed. Regardless of the outcome of the three blocks, all patients were offered nerve surgery. A permanent positive effect on the pain following surgery was seen in 7/16 (44%) of the patients. No beneficial effect or an increase was reported by 9/16 (56%) of the patients. Five of the 16 patients (31%) were (as good as) pain free, and 2 (12%) had partial pain reduction. In 6/16 (38%) there was no effect of the surgery, whereas a temporary pain reduction was seen in 2 (12%) of the patients and an increase in 1 (6%).

Between 2008 and 2013, a total of 24 patients (11 male, 13 female) met the inclusion criteria. (Fig 3) The mean age at first consultation was 43 years (median 39, SD 14). The mean interval between the nerve lesion and the first block was 40 months (median 15, SD 20). The sensory nerves involved were located in the lower extremity in 18 patients (75%), in the upper extremity in 4 (16%) patients and at the thoracic level in 2 (8%) (Table 1, S1 Minimal Data Set).

**Table 1:** Effect of blocks and surgery on pain.

Patient	Nerve	Age	Interval Block 1-lesion	Block 1 Lidocaïne	Block 2 Saline	Block 3 Lidocaïne	Result surgery
1	superficial radial	62	4	1	0	1	0
2	cutaneous lateral femoral	15	69	3	2	3	0
3	saphenous	70	20	0	0	0	0
4	infrapatellar	35	25	1	2	3	0
5	infrapatellar	28	62	2	0	0	0
6	distal tibial	38	15	0	3	0	0
7	superficial radial	40	57	2	3	2	1
8	sural	63	38	0	3	0	1
9	intercostobrachial	37	45	0	3	0	2
10	palmar digital II	44	20	1	3	1	2
11	medial plantar	49	6	0	0	3	2
12	cutaneous lateral femoral	36	59	2	2	0	2
13	cutaneous superficial peroneal	61	19	2	0	0	2
14	cutaneous superficial peroneal	34	23	1	3	1	3
15	cutaneous superficial peroneal	50	23	2	0	2	3
16	infrapattelar	32	32	1	3	1	4
17	intercostal 4	54	9	2	2	2	*
18	dorsal branch ulnar	38	9	2	2	2	*
19	calcaneal and medial plantar	20	16	2	0	2	*
20	cutaneous posterior femoral	36	33	0	2	3	**
21	infrapatellar	42	290	1	3	1	**
22	cutaneous deep peroneal	50	12	0	0	0	**
23	cutaneous superficial peroneal	37	6	2	0	0	**
24	sural	60	15	1	2	3	**

Age at nerve lesion in years. Interval block 1 and lesion in months. Effects of the lidocaine or saline injection: 0) No effect; 1) Pain relief for several hours; 2) Pain relief for days or permanent pain relief; 3) Increase of pain for hours or days. Result of surgery: 0) no effect, 1) partial pain reduction, 2) (as good as) pain free, 3) temporary pain reduction, 4) increase of pain as compared to the pre-operative level.

* Permanent pain relieving effect as response to the block

** Refrained from surgery

The pain started after a surgical intervention in 18/24 of the patients (75%), of which the pain developed immediately following surgery in 13/16 (80%), and in 3/16 (20%) of the patients after weeks to months. In 6/24 (25%) of patients, the pain started following a trauma; in 3 immediately following trauma, and in the other 3 patients weeks to months later.

### Effect of nerve blocks

The different effects of the blocks on pain are shown in Table 1 and summarized in Table 2.

**Table 2:** Summary of the different effects of the three blocks on pain.

Blocks	Lidocaine 1 (n = 24)	Lidocaine 2^#^ (n = 24)	Lidocaine 1+2 (n = 48)	Saline (n = 24)	Total (n = 72)
No effect	7 (29)	9 (38)	16 (33)	9 (38)	25 (35)
Relief for several hours	7 (29)	5 (21	12 (25)	0	12 (17)
Relief for days or permanent	9 (38)	5 (21)	14 (29)	7 (29)	21 (29)
Any relief	16 (66)	10 (42)	26 (54)*	7 (29)	33 (46)
Increase for hours or days	1 (4)	5 (21)	6 (13)	8 (33)	14 (19)

Percentages are between brackets.

^#^ The effect of the second lidocaine block was similar to the first in 16/24 (66%).

* Pain reduction following both lidocaine blocks was found in 10/24 (42%) of the patients.

In 26 of the 48 lidocaine blocks (54%) a pain reducing effect was observed. The effect of the second lidocaine block was similar to the first in 16/24 (66%). Pain reduction following both lidocaine blocks was found in 10/24 (42%) of the patients.

A pain relieving effect of the first lidocaine block was obtained in 16/24 (66%) of the patients, which lasted for several hours in 7/24 (29%) and for many days in 9/24 (38%). There was no effect of the first lidocaine block in 7/24 (29%) of the patients. One patient of the 24 (4%) observed more pain following the first lidocaine block and 5/24 (21%) after the second lidocaine block. In total, 16/24 (66%) of the patients had strong pain reduction in at least one of the two lidocaine blocks regardless of the duration of the effect.

A pain relieving effect of placebo lasting for days was noted in 7/24 (29%) of the blocks. No effect on pain following saline injection was noted in 9/24 (38%) of patients. A temporary increase of pain following saline injection was seen in 8/24 (33%).

No permanent adverse effects of the injections were seen. In 3 of the 24 patients, the injections induced a continuous pain relieving effect to such an extent that these patients did not need surgery nor did they require further pain treatment. Five of the remaining 21 patients who were offered surgery refrained. They requested a 100% guarantee to get significant pain relief following surgery which could not be given, or nerve surgery was judged to be too radical.

### Nerve surgery

Sixteen of the 21 patients opted for surgery. The mean interval between the nerve lesion and the operation for pain was 38 months (Median 27, SD 20). The mean interval between the first block and the operation was 6 months (Median 5, SD 3). The mean follow-up after the operation was 15 months (Median 11, SD 13).

**Fig 4:**
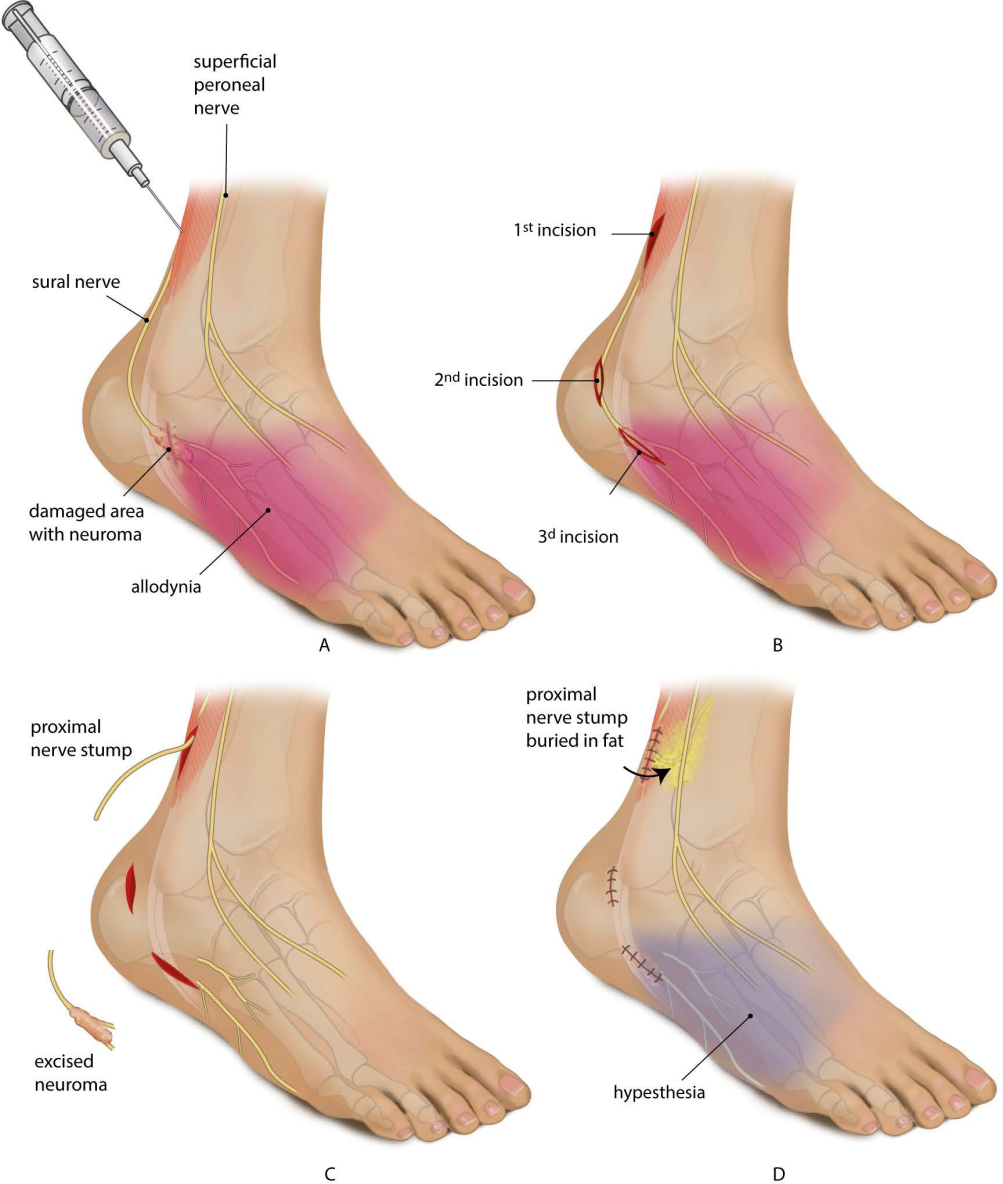
Case illustration of a 63 year patient (number 8, Table 1) in whom blocking a nerve had no pain relieving effect, but nerve surgery had. Following the current selection algorithm, this patient would not have been operated. The patient had complaints befitting ankle arthrosis. An isolated subtalar arthrodesis was performed by placing compression screws. Immediate postoperative, the patient had severe neuropathic pain with allodynia in the sural nerve area limiting the walking distance to around 150 meters. Conservative treatment failed. A: At inspection 38 months after the onset of the pain, a scar of the screw placement was seen around 4 centimetres below the lateral malleolus. Percussion in the scarred damaged area provoked irradiating painful sensations in the sural nerve area. The sural nerve was blocked three times in 4 weeks (with a 2-week interval between injections) consisting of two single-blind injections with a volume of 4 cc. lidocaine 1% and one with placebo. There was no effect of the lidocaine injections and following saline injection the pain increased temporarily. B: At surgery, the sural nerve was identified in undamaged area (1^st^ incision) and dissected free subcutaneously. A second incision was made and the sural nerve was followed distally towards the scar. Subsequently, a third incision was made over the scar and a damaged sural nerve was identified. C: The damaged sural nerve was cut and the abnormal looking nerve tissue was resected. Pathological examination of the abnormal tissue showed traumatic neuroma. The resection plane of the proximal stump showed normal myelinated fibers and fascicles. D: The proximal stump of the sural nerve was loosely buried in fat proximal to the ankle joint. Postoperatively, his pain decreased significantly. The area with allodynia disappeared and became hypesthetic. The patient could walk again for at least one hour.

At surgery, the nerve was cut and buried in fat.(Fig 4) A permanent positive effect on the pain following surgery was seen in 7/16 (44%) of the patients.(Table 1) No beneficial effect or an increase was reported by 9/16 (56%) of the patients. Five of the 16 patients (31%) were (as good as) pain free, and 2 (12%) had partial pain reduction. In 6/16 (38%) there was no effect of the surgery, whereas a temporary pain reduction was seen in 2 (12%) of the patients and an increase in 1 (6%).

### The predictive ability of the nerve block on the outcome of surgery

**Fig 5:**
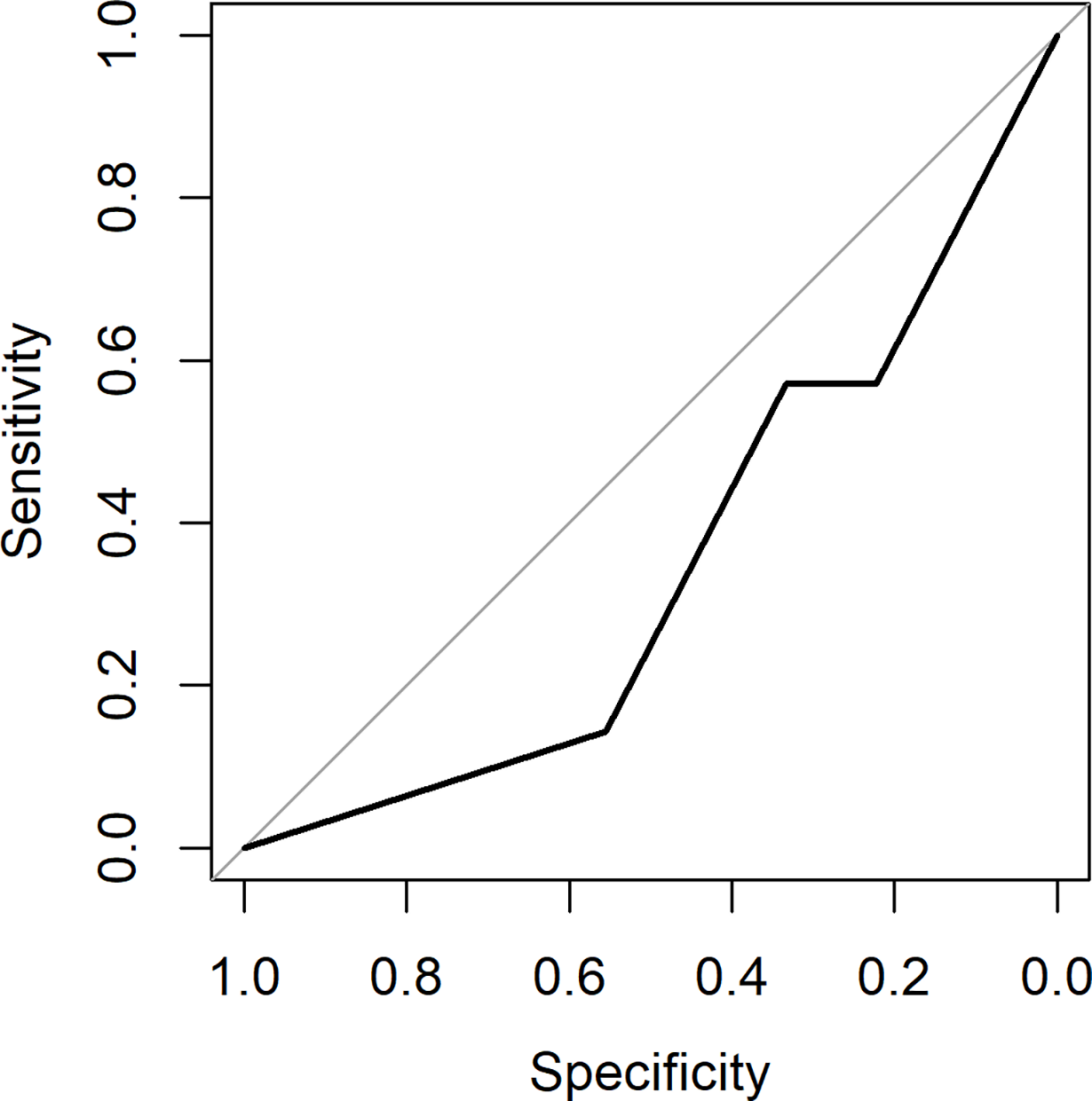
The area under the Receiver Operating Curve (AUC) of the first lidocaine block. The AUC was 0.35 with a 95% confidence interval (CI) from 0.077 to 0.62. The AUC is less than 0.62 at 95% confidence (two-sided), and hence the predictive ability of the lidocaine nerve block is poor.

The AUC of the first lidocaine block was 0.35 with a 95% confidence interval (CI) from 0.077 to 0.62.(Fig 5) We conclude at 95% confidence (two-sided) that the AUC is less than 0.62, and hence that the predictive ability of the lidocaine nerve block is poor.

**Fig 6:**
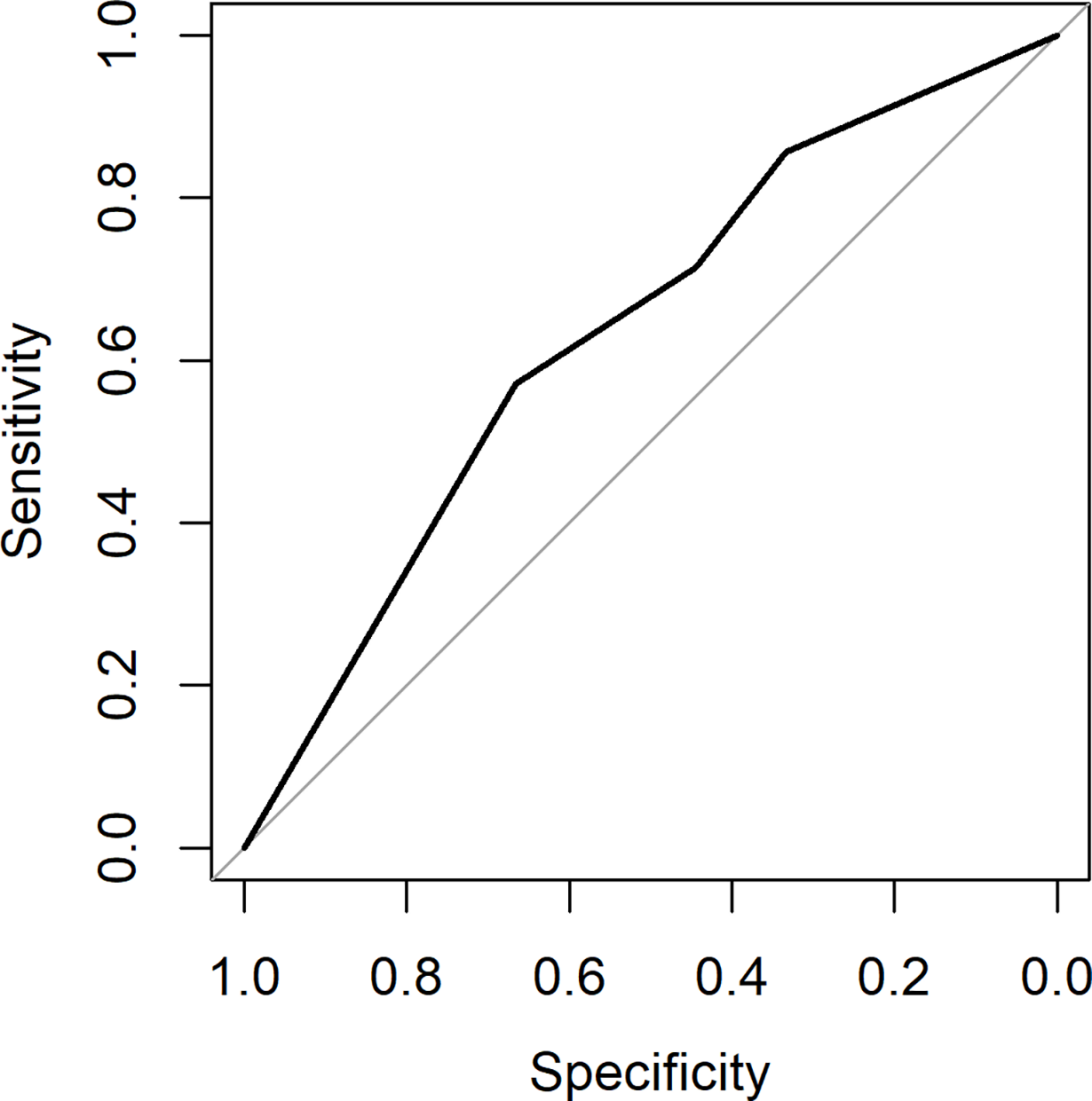
The AUC of the second lidocaine block was 0.63 (95% CI 0.36 to 0.9).

**Fig 7:**
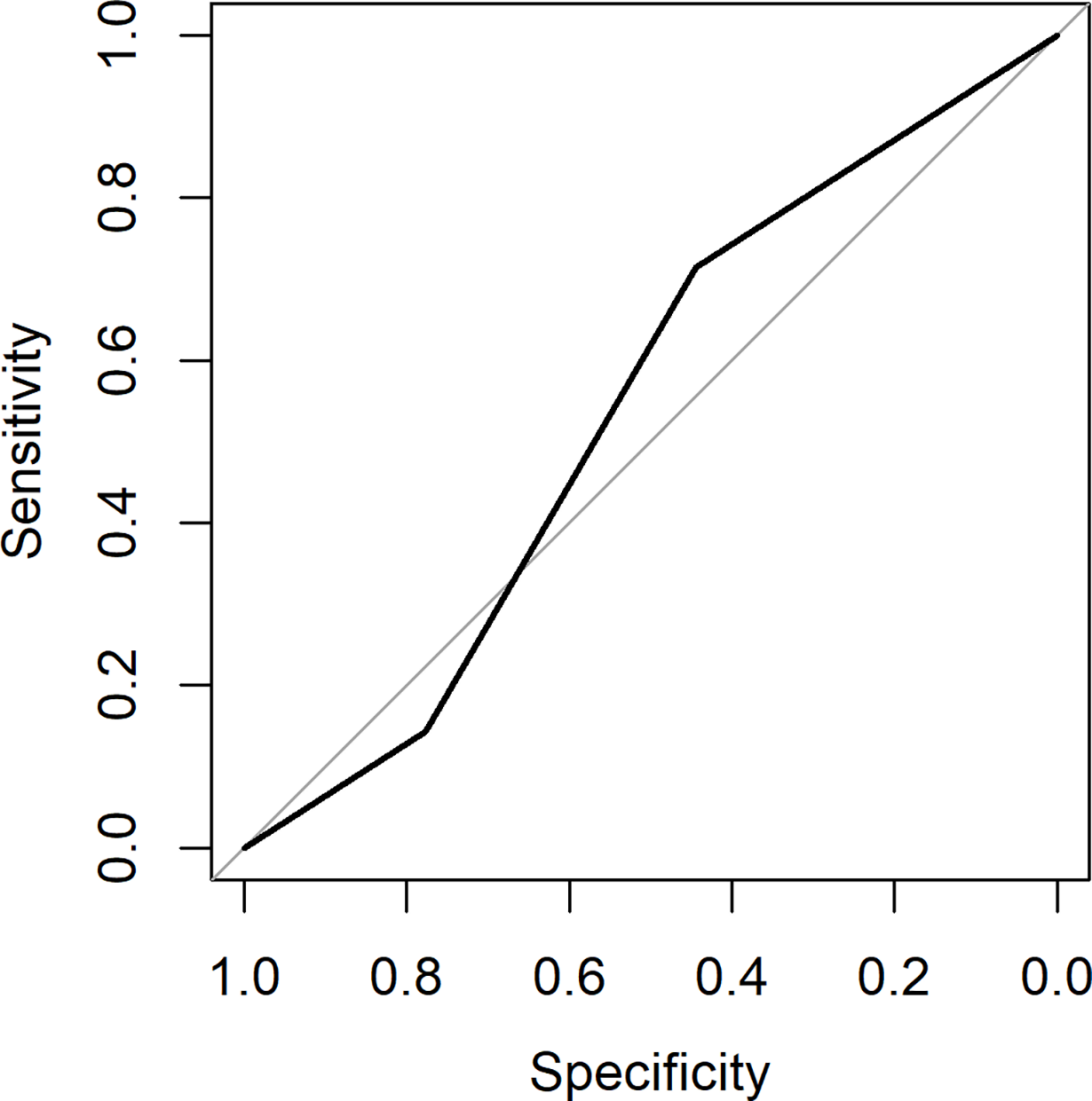
The AUC of the saline block was 0.53 (95% CI: 0.26–0.82).

The AUC of the second lidocaine block was 0.63 (95% CI 0.36 to 0.91, Fig 6) and of the saline block 0.53 (95% CI: 0.26–0.82, Fig 7). There was no difference between the first and second lidocaine block (p = 0.24). The outcome of the second lidocaine block and saline block does not change the conclusion of the first block.

## Discussion

Satisfactory control of neuropathic pain following nerve injury can be extremely difficult to obtain. Nerve surgery is occasionally considered when conservative measures fail.[6] Diagnostic blocks are used to select patients who might benefit from nerve surgery.[18,52] (Fig 1) The surgery entails neuroma resection and burying of the proximal stump in an area of healthy tissue in such a way that mechanical strain or pressure forces on the stump cannot occur. (Fig 4)

Usually one lidocaine block is given in an un-blinded setting when selecting patients for surgery.[18] (Fig 1) Our block protocol for patients with persistent postsurgical or nerve injury induced probable neuropathic pain[2] consisted of three injections including a placebo. In addition, the patient was blinded. Nerve surgery to reduce pain was offered to all patients, regardless of the outcome of the three blocks. The predictive ability of the lidocaine block in this study was poor. Based on these results we conclude that block responses for selection for surgery should be carefully interpreted as they may not be as predictive as generally presumed. Actually, the use of blocks to select patients for surgery should be critically appraised.

Three technical factors potentially affect the outcome of our analysis: the blocking procedure, the surgery and the blinding. First, we may actually not have injected lidocaine in the close surroundings of the involved nerve. This would then erroneously lead to false negative blocks. Our blocks were performed following current routine. We injected 4 cc. of lidocaine to reduce the chance of lidocaine not reaching the nerve. Usually, a volume of 1–2 cc. is used.[19,20,29,30,39,50,66] We think that this factor as such, cannot explain the low predictive value. Recently, it has become possible to directly visualize and identify small nerves with high-resolution ultrasound.[67,68] Whether application of this technique might contribute to optimizing the blocking procedure, and thereby patient selection for surgery, has yet to be established.

Second, there is no consensus as to which envelopment of the proximal stump is optimal. (Fig 4) The proximal stump can be buried in bone[10,14–16,33,43,48,51], muscle,[9,10,12,14–16,18,21–29,33,35,41,46–48,51,52,60,66] vein[12,32,36–38] or nerve.[13,57,59] Local factors in the area of the damaged nerve play a role in neuropathic pain[69] and provocation might not be entirely a mechanical problem.[70] A relative excess of nerve growth factor (NGF) in the surrounding tissue may also play a role.[71] Differences in NGF content between muscle and fat are likely to exist.[72,73] Like others we routinely buried the proximal stump in fat.[55,74] Envelopment of the nerve stump in muscle[75] might give a different effect on pain reduction which will inherently change the predictive value of a block. Given the low values found in our study, it seems unlikely that the effect of envelopment will be of a sufficient magnitude to increase the value of blocking.

Third, in routine practice (Fig 1) patients are frequently told that a local anaesthetic will be given whereas we blinded the patient for the content of the block. We cannot assess the power of the effect of positive expectations because our patients were blinded and we did not include blocks where we revealed the potential working mechanism. It is possible that responses differ if blocks are given in an un-blinded fashion. Whether revealing the content will sufficiently raise the predictive ability of the block to make it valuable is questionable. The effect of saline could be placebo,[76] but also real and based on e.g. diluting local substances involved in the inflammatory response of neuropathic pain. The effect of saline differed in varying degrees from that of lidocaine. Multiple reasons may account for this effect, either separately or in combination. It requires further studies to elucidate this phenomenon. In itself, however, it does not conflict with our findings.

The low accuracy of blocks to predict the effect of surgery probably reflects the multifactorial complex nature and origin of neuropathic pain. Following the nerve injury, the axonopathy and demyelination trigger membrane remodelling in injured afferents and in uninjured neighbours supplying the affected region. Subsequently, cellular excitability increases in part due to sodium (Na+)- channel dysfunction. This leads to ectopic generation of action potentials constituting a primary neuropathic pain signal.[77] Lidocaine inhibits Na+ channels preventing the propagation of action potentials.[78] Theoretically, a Na+ channel-blocking agent should therefore help to relieve neuropathic pain when the impulse source is at or distal to the site of application. In our study, two-third of the patients experienced a strong pain reduction in at least one of the two lidocaine blocks. Pain reduction following both lidocaine blocks was only found in less than half of the patients.

Generally, the duration of the pain relieving effect of infiltration with 1% lidocaine is two to six hours.[79] In our study, the effect varied between several hours and days. Some patients even experienced permanent pain reduction and further treatment was not required. An explanation for these varying effects is difficult to provide. Permanent effects have been observed by others as well.[80]

The varying effect of lidocaine in terms of the duration and intensity indicate that not only Na+- channel dysfunction is at play. Multiple different sites for pain along the neural axis are involved.[69] Central sensitization may have developed since many patients were referred late potentially reducing the effect of peripheral treatment.

Lidocaine is used in the vast majority of the blocks.[7,9,10,12,19–23,29,30,36–39,42,45,46,48–50,52,56,57,60,61,66] It is not known whether a block with a different local anaesthetic, like Bupivacaine[13] with a longer duration of action may increase the accuracy of prediction.

Pain reduction can be achieved with nerve surgery, but to what extent and in which percentage of the patients cannot be summarized from the literature because of the different ways of reporting. In this series, 44% of the patients had good pain relief. Traumatic neuromas show spontaneous discharge activity and ectopic sensitivity to mechanical stimuli.[81] It might be the reduction of these phenomena that underlies the beneficial effect of resection. Strictly speaking, however, part of the effect of surgery might even be placebo.[82]

Immediate surgical repair of the damaged nerve would be optimal, but usually requires a graft. This would then imply sacrificing an intact cutaneous nerve, most often the sural nerve, in order to restore the damaged one. This trade-off is generally considered not favourable enough to justify grafting. Whether biodegradable nerve tubes[83] or cadaveric nerve grafts[84] can serve as an alternative for autologous grafts for this indication has not yet been systematically studied.

The strength of our study is that we only included patients who met the criteria for persistent postsurgical or nerve injury induced probable neuropathic pain.[2] Additionally, the patient was blinded, the blocks were repeated three times and the time course of the effect was noted. Finally, surgery was also offered when the lidocaine blocks were negative. We thereby bypassed a selection bias, which is present when surgery is only carried out in patients whom had a positive block.

We recognize several weaknesses in this study. Ideally, a prospective randomized double blinded set-up would be best. Furthermore, a relative small group of patients was surgically treated. The predictive ability of the block, however, was so low that the possibility that it would be improved by the inclusion of a larger number of patients is small. In our study, 16 different anatomical nerves were damaged, all to a different extent. The penetration of lidocaine might have been influenced by the relative contribution of supportive tissue, the fiber composition, number of fibers and fascicles and fascicular pattern which varies between nerves and individuals.[85] Also, the interval between trauma, block and surgery may have consequences for the response due to central sensitisation. Finally, we did not use questionnaires (such as the McGill one), or non-language based scales (such as the Visual Analogue Score) to document the effect of the block because all have considerable limitations.[86] The VAS score does only provide information about the amount of pain, but not on the duration of the relief. We documented the effect on pain in those categories that are clinically encountered taking both the direction as well as the duration into account.

Offering surgery to patients with focal nerve injury and neuropathic pain only if a pain relieving response to one open block is obtained is probably not a good method. Open blocks may not be as predictive as generally presumed. The selection algorithm needs to be critically re-evaluated.

## Supporting information

S1 Minimal Data Set (DOCX)
