# Supplementary material for: Predictive value of a diagnostic block in focal nerve injury with neuropathic pain when surgery is considered
Source: PLoS One. 2018 Sep 12;13(9):e0203345. doi: 10.1371/journal.pone.0203345 (PMC6135496; doi:10.1371/journal.pone.0203345)
Supplement: S1 Minimal Data Set — (DOCX) [file pone.0203345.s001.docx]

Minimal Data Set

Effect of blocks and surgery on pain

| Patient | Nerve | Age | Interval Block 1-lesion | Block 1  Lidocaïne | Block 2  Saline | Block 3  Lidocaïne | Result surgery |
| --- | --- | --- | --- | --- | --- | --- | --- |
| 1 | superficial radial | 62 | 4 | 1 | 0 | 1 | 0 |
| 2 | cutaneous lateral femoral | 15 | 69 | 3 | 2 | 3 | 0 |
| 3 | saphenous | 70 | 20 | 0 | 0 | 0 | 0 |
| 4 | infrapatellar | 35 | 25 | 1 | 2 | 3 | 0 |
| 5 | infrapatellar | 28 | 62 | 2 | 0 | 0 | 0 |
| 6 | distal tibial | 38 | 15 | 0 | 3 | 0 | 0 |
| 7 | superficial radial | 40 | 57 | 2 | 3 | 2 | 1 |
| 8 | sural | 63 | 38 | 0 | 3 | 0 | 1 |
| 9 | intercostobrachial | 37 | 45 | 0 | 3 | 0 | 2 |
| 10 | palmar digital II | 44 | 20 | 1 | 3 | 1 | 2 |
| 11 | medial plantar | 49 | 6 | 0 | 0 | 3 | 2 |
| 12 | cutaneous lateral femoral | 36 | 59 | 2 | 2 | 0 | 2 |
| 13 | cutaneous superficial peroneal | 61 | 19 | 2 | 0 | 0 | 2 |
| 14 | cutaneous superficial peroneal | 34 | 23 | 1 | 3 | 1 | 3 |
| 15 | cutaneous superficial peroneal | 50 | 23 | 2 | 0 | 2 | 3 |
| 16 | infrapattelar | 32 | 32 | 1 | 3 | 1 | 4 |
| 17 | intercostal 4 | 54 | 9 | 2 | 2 | 2 | * |
| 18 | dorsal branch ulnar | 38 | 9 | 2 | 2 | 2 | * |
| 19 | calcaneal and medial plantar | 20 | 16 | 2 | 0 | 2 | * |
| 20 | cutaneous posterior femoral | 36 | 33 | 0 | 2 | 3 | ** |
| 21 | infrapatellar | 42 | 290 | 1 | 3 | 1 | ** |
| 22 | cutaneous deep peroneal | 50 | 12 | 0 | 0 | 0 | ** |
| 23 | cutaneous superficial peroneal | 37 | 6 | 2 | 0 | 0 | ** |
| 24 | sural | 60 | 15 | 1 | 2 | 3 | ** |

Legend: Age at nerve lesion in years. Interval block 1 and lesion in months. Effects of the lidocaine or saline injection: 0) No effect; 1) Pain relief for several hours; 2) Pain relief for days or permanent pain relief; 3) Increase of pain for hours or days. Result of surgery: 0) no effect, 1) partial pain reduction, 2) (as good as) pain free, 3) temporary pain reduction, 4) increase of pain as compared to the pre-operative level.

* Permanent pain relieving effect as response to the block; ** Refrained from surgery
